# Trends and Efficacy of Interferon-Free Anti–hepatitis C Virus Therapy in the Region of High Prevalence of Elderly Patients, Cirrhosis, and Hepatocellular Carcinoma: A Real-World, Nationwide, Multicenter Study of 10 688 Patients in Japan

**DOI:** 10.1093/ofid/ofz185

**Published:** 2019-04-15

**Authors:** Hidenori Toyoda, Masanori Atsukawa, Haruki Uojima, Akito Nozaki, Hideyuki Tamai, Koichi Takaguchi, Shinichi Fujioka, Makoto Nakamuta, Toshifumi Tada, Satoshi Yasuda, Makoto Chuma, Tomonori Senoh, Akemi Tsutsui, Naoki Yamashita, Atsushi Hiraoka, Kojiro Michitaka, Toshihide Shima, Takehiro Akahane, Ei Itobayashi, Tsunamasa Watanabe, Hiroki Ikeda, Etsuko Iio, Shinya Fukunishi, Toru Asano, Yoshihiko Tachi, Tadashi Ikegami, Kunihiko Tsuji, Hiroshi Abe, Keizo Kato, Shigeru Mikami, Hironao Okubo, Noritomo Shimada, Toru Ishikawa, Yoshihiro Matsumoto, Norio Itokawa, Taeang Arai, Akihito Tsubota, Katsuhiko Iwakiri, Yasuhito Tanaka, Takashi Kumada

**Affiliations:** 1 Department of Gastroenterology, Ogaki Municipal Hospital, Japan; 2 Department of Internal Medicine, Division of Gastroenterology and Hepatology, Nippon Medical School, Tokyo, Japan; 3 Department of Gastroenterology, Internal Medicine, Kitasato University School of Medicine, Sagamihara, Japan; 4 Gastroenterology Center, Yokohama City University Medical Center, Japan; 5 Department of Hepatology, Wakayama Rosai Hospital, Japan; 6 Department of Hepatology, Kagawa Prefectural Central Hospital, Takamatsu, Japan; 7 Department of Gastroenterology, Okayama Saiseikai General Hospital, Japan; 8 National Hospital Organization Kyushu Medical Center, Fukuoka, Japan; 9 Gastroenterology Center, Ehime Prefectural Central Hospital, Matsuyama, Japan; 10 Department of Gastroenterology and Hepatology, Saiseikai Suita Hospital, Japan; 11 Department of Gastroenterology, Japanese Red Cross Ishinomaki Hospital, Japan; 12 Department of Gastroenterology, Asahi General Hospital, Japan; 13 Department of Internal Medicine, St. Marianna University School of Medicine, Kawasaki, Japan; 14 Department of Virology and Liver Unit, Nagoya City University, Graduate School of Medical Sciences, Japan; 15 Second Department of Internal Medicine, Osaka Medical College, Japan; 16 Department of Internal Medicine, Division of Gastroenterology and Hepatology, Tokyo Metropolitan Bokuto Hospital, Japan; 17 Department of Gastroenterology and Hepatology, Komaki City Hospital, Japan; 18 Tokyo Medical University Ibaraki Medical Center, Japan; 19 Center for Gastroenterology, Teine Keijinkai Hospital, Sapporo, Japan; 20 Department of Internal Medicine, Division of Gastroenterology and Hepatology, Shinmatusdo Central General Hospital, Matsudo, Japan; 21 Department of Internal Medicine, Division of Gastroenterology, Kikkoman General Hospital, Noda, Japan; 22 Department of Gastroenterology, Juntendo University Nerima Hospital, Tokyo, Japan; 23 Department of Internal Medicine, Division of Gastroenterology and Hepatology, Otakanomori Hospital, Kashiwa, Japan; 24 Department of Hepatology, Saiseikai Niigata Daini Hospital, Japan; 25 Department of Gastroenterology and Hepatology, Jikei University School of Medicine Kashiwa Hospital, Japan; 26 Department of Internal Medicine, Division of Gastroenterology, Nippon Medical School Chiba Hokusoh Hospital, Inzai, Japan; 27 Core Research Facilities for Basic Science, Jikei University School of Medicine, Tokyo, Japan

**Keywords:** chronic HCV infection, direct-acting antivirals, exact matching, hepatocellular carcinoma

## Abstract

**Background:**

We investigated changes in patient characteristics, rate of sustained virologic response (SVR), and factors associated with SVR after anti-hepatitis C virus (HCV) therapy with direct-acting antiviral (DAA) regimens in real-world practice in Japan, where patients with HCV are characterized by older age and high prevalence of cirrhosis and hepatocellular carcinoma (HCC).

**Methods:**

Changes in patient characteristics and SVR rates were evaluated from medical records among 10 688 patients who started interferon (IFN)-free DAA therapy between September 2014 and June 2018 in a nationwide, multicenter study. Factors associated with failure of SVR were analyzed. In particular, effects of cirrhosis or history of HCC on SVR were assessed by exact matching.

**Results:**

Patient age was becoming younger and baseline liver fibrosis was becoming milder over time. Overall SVR rate was 95.4%. The SVR rates increased over time in patients without a history of IFN-free DAA therapy. Multivariate analysis revealed that cirrhosis was unfavorably associated with achievement of SVR in both patients with genotype 1 (odds ratio, 1.68; 95% confidence interval [CI], 1.27–2.21) and genotype 2 (odds ratio, 1.69; 95% CI, 1.01–2.78). Comparisons after exact matching showed that the SVR rate was significantly lower in patients with cirrhosis than without it, whereas patients with and without a history of HCC had similar SVR rates.

**Conclusions:**

Background characteristics of patients who undergo IFN-free DAA therapy are changing in Japan. Patients without a history of IFN-free DAA therapy have high SVR rates. Exact matching confirmed that cirrhosis significantly influences the achievement of SVR in real-world settings.

Hepatitis C virus (HCV) infection is a serious health problem that currently affects more than 180 million people around the world [1]. It is associated with severe liver complications such as cirrhosis and hepatocellular carcinoma (HCC). Therapy to eradicate HCV is an essential treatment for chronic HCV infection that reportedly prevents the progression of liver fibrosis [2–4] and the development of HCC [5–7], resulting in decreased all-cause mortality [8, 9]. The landscape of antiviral therapy for HCV dramatically changed with the emergence of interferon (IFN)-free direct-acting antiviral (DAA) agent regimens capable of producing a sustained virologic response (SVR), ie, the eradication of HCV, in more than 90%–95% of patients. In addition, the high tolerability of these IFN-free regimens broadened the range of potential candidates for anti-HCV therapy. These regimens can be used in patients with advanced age [10, 11], advanced fibrosis or cirrhosis [12], or renal impairment [13, 14].

In Japan, patients with HCV infection are characterized by the older age and high prevalence of patients complicated with cirrhosis or HCC [15, 16]. Hepatitis C virus infection was considered to be widespread due to the temporal trend of drug use just after World War II and, afterward, by medical procedure, transfusion, or folk remedies [17]. There is an established national public insurance system that applies for all peoples living in Japan. In addition, there is an additional national support system for the treatment of viral hepatitis, with which patients can undergo anti-HCV DAA therapy with low cost: the same price regardless of DAA-regimen. Multiple IFN-free DAA regimens were approved by Japanese government and have been in clinical use since September 2014. All of these regimens were approved based on preapproval clinical trials conducted in Japan [18–26], all of which had shown high SVR rates and tolerability. However, the real-world use and efficacy for these regimens have not been sufficiently demonstrated. Although IFN-free DAA regimens have much higher SVR rates than IFN-based regimens, differences in SVR rates between DAA regimens and the factors associated with failure to achieve SVR, which remains even in the era of DAA therapy, have not been fully described. In addition, changes in the background characteristics of patients who undergo DAA therapy after approval, which will vary across countries, has not been analyzed.

In this study, we investigated the real-world trends of IFN-free DAA therapy and their efficacy in Japan, with 10 688 patients with chronic HCV infection in a nationwide, multicenter study.

## PATIENTS AND METHODS

This nationwide, multicenter study included 27 institutions across Japan, located in the Hokkaido, Tohoku, Kanto, Hoku-Shinetsu, Chubu, Kinki, Chugoku, Shikoku, and Kyushu areas (Supplementary Figure 1). All patients underwent IFN-free DAA regimens for HCV infection between September 2014 and June 2018. Each regimen was selected after approval by Japanese government and became available in a real-world setting. In all patients, treatment was managed in the hepatology section of each hospital by certified hepatologists, including patient evaluation, selection of regimen, treatment, and follow-up. Persistent HCV infection before DAA therapy was documented in all patients by both positive serum HCV antibody titers and serum HCV ribonucleic acid (RNA) measured using a real-time polymerase chain reaction (PCR)-based method. Hepatitis C virus genotype was assessed using PCR with genotype-specific primers. Patients coinfected with hepatitis B virus or human immunodeficiency virus and patients with other liver diseases, including autoimmune hepatitis and primary biliary cholangitis, were excluded from the study.

All patients underwent IFN-free DAA therapy chosen based on HCV genotype. Patients infected with HCV genotype 1 underwent one of the following regimens: daclatasvir and asunaprevir (DCV-ASV) for 24 weeks; ledipasvir and sofosbuvir (LDV-SOF) for 12 weeks; ombitasvir, paritaprevir, and ritonavir (OMV-PRV-Rit) for 12 weeks; elbasvir and grazoprevir (EBR-GPR) for 12 weeks; daclatasvir, asunaprevir, and beclabuvir (DCV-ASV-BCV) for 12 weeks; or glecaprevir and pibrentasvir (GLE-PIB) for 8 or 12 weeks. Patients infected with HCV genotype 2 underwent one of the following regimens: sofosbuvir and ribavirin (SOF-RBV) for 12 weeks; ombitasvir, paritaprevir, ritonavir, and ribavirin (OMV-PRV-Rit-RBV) for 16 weeks; or GLE-PIB for 8 or 12 weeks. In patients with HCV genotype 1, it was known by clinical trials [18, 21] that resistance-associated substitutions (RASs) in HCV-nonstructural protein 5A (NS5A) region unfavorably influenced on the achievement of SVR by DCV-ASV or OMV-PRV-Rit regimen, and it was not recommended for patients with these RASs, although the measurement of these RASs is not covered by insurance.

In all patients, baseline background characteristics were collected from medical records, including age, gender, HCV genotype, platelet count, aspartate aminotransferase (AST), alanine aminotransferase (ALT), FIB-4 index, AST-platelet ratio index (APRI), presence of cirrhosis, history of HCC, history of IFN-based therapy, history of IFN-free therapy, IFN-free DAA regimen used, and therapy start date. In more than half of the patients with HCV genotype 1 infection, the presence of known RASs in the HCV-NS5A region, ie, amino acid residues 31 and 93, was assessed before the start of therapy using direct sequencing [27]. The presence of cirrhosis was assessed clinically by an attending hepatologist based on imaging and endoscopic findings, including the presence of esophageal or gastric varices, collateral veins due to portal hypertension, and splenomegaly. Patients with compensated cirrhosis were included in the study, but patients with decompensated cirrhosis, diagnosed based on the presence of ascites, hepatic encephalopathy, variceal bleeding, or jaundice, were not included because IFN-free DAA therapy is not allowed in this population during the study period in Japan. In addition, only patients with inactive HCC that were treated with curative intent were included, because IFN-free DAA therapy is not allowed for patients with active HCC. Two laboratory liver fibrosis indices, FIB-4 index [28] and APRI [29], were calculated based on the following formulas: FIB-4 index = AST [IU/L] × age [years]/platelet count [10^9^/L] × ALT [IU/L]^1/2^; and APRI = (AST [IU/L]/upper limit of normal AST [IU/L]) × 100/platelet count [10^9^/L].

Regarding treatment outcomes, SVR was defined as undetectable serum HCV RNA based on a real-time PCR-based assay at 12 weeks after the end of treatment.

The protocol of this multicenter study was in compliance with the Helsinki Declaration and was approved by each participating institution. Written informed consent was waived due to the retrospective nature of this study.

### Statistical Analysis

Baseline characteristics and SVR rates between patient subgroups and differences in distribution were analyzed using the χ^2^ test. Differences in quantitative values were analyzed using the Mann-Whitney *U* test. The Cochran-Armitage test or Jonckheere-Terpstra test was used to analyze associations between therapy start month, grouped into a 6-month interval, and patient background characteristics or SVR rate. Univariate and multivariate logistic regression were performed to identify factors associated with SVR. Variables that reached statistical significance (*P* < .05) in the univariate analysis were included in the multivariate analysis. Statistical analysis was performed using JMP statistical software, version 6.0 (Macintosh version; SAS Institute, Cary, NC). All *P* values were derived from 2-tailed tests, with *P* < .05 accepted as statistically significant.

To accurately compare SVR rates between patients with and without cirrhosis, we conducted exact matching via one-to-one pairing of patients without replacement based on patient age, gender, history of IFN-based therapy, history of IFN-free DAA therapy, history of HCC, treatment regimen, HCV genotype, and presence of HCV-NS5A-RASs in patients with HCV genotype 1 infection. Likewise, for accurate comparisons of SVR rates between patients with and without a history of HCC, we conducted exact matching via one-to-one pairing of patients without replacement based on patient age, gender, history of IFN-based therapy, history of IFN-free DAA therapy, presence of cirrhosis, treatment regimen, HCV genotype, and presence of HCV-NS5A-RASs in patients with HCV genotype 1 infection. Exact matching was performed using SPSS, version 18.0 (IBM, Tokyo, Japan).

## RESULTS

### Background Characteristics of Patients Who Underwent Interferon-Free Direct-Acting Antiviral Therapy

Table 1 shows the background characteristics of the study patients. All patients were of Japanese ethnicity. The median age was 68 years (interquartile range, 59–75) and 51.2% were female. The percentage of patients with a history of IFN-based therapy and IFN-free therapy was 27.1% and 3.1% (Supplementary Table 1), respectively. A total of 27.9% of patients had compensated cirrhosis, and 12.4% of patients had a history of HCC treated with curative intent. A total of 72.1% of patients were infected with HCV genotype 1, the predominant genotype in Japan [15]. Among 5107 of 7706 patients (66.3%) infected with HCV genotype 1 in whom HCV-NS5A RAS status was assessed, wild-type HCV was found in 4118 patients (80.6%). The remaining 989 (19.4%) had RASs (ie, amino acid mutation at residue 31, or 93, or both positions). Whereas 16.4% (804 of 4880) of patients without a history of IFN-free DAA therapy had HCV with RASs, 81.5% (185 of 227) of patients with a history of previous IFN-free DAA therapy had RASs. The percentage with NS-5A RASs was 6.7% in patients treated with DCV-ASV, 36.8% in patients treated with LDV-SOF, 4.3% in patients treated with OMV-PRV-Rit, 21.5% in patients treated with EBR-GPR, 57.6% in patients treated with DCV-ASV-BCV, and 45.0% in patients treated with GLE-PIB. Because SVR rates were low in patients with NS-5A RASs when treated with DCV-ASV or OMV-PRV-Rit in preapproval clinical trials [18, 21], most patients with baseline NS-5A RASs did not receive these regimens. Among 1587 patients infected with HCV genotype 2 in whom sub-genotype information was available, HCV genotype 2a was observed in 62.3% of patients treated with SOF-RBV, 91.2% of patients treated with OMV-PRV-Rit-RBV, and 63.1% of patients treated with GLE-PIB. Because a low SVR rate was reported in patients with HCV genotype 2b when treated with OMV-PRV-Rit-RBV [22], patients treated with this regimen predominantly had HCV genotype 2a infection. Changes in background characteristics of patients and SVR rates over time are described in Supplementary Material.

**Table 1.** Baseline Characteristics of the Study Patients (N = 10 688)

Age (years)	68 (59–75)
Gender (male/female)	5140 (48.1)/5548 (51.9)
History of interferon-based therapy (no/yes)	7791 (72.9)/2897 (27.1)
History of interferon-free DAA therapy (no/yes)	10352 (96.9)/336 (3.1)
Cirrhosis^a^ (absent/present)	7711 (72.1)/2977 (27.9)
History of hepatocellular carcinoma treated with curative intent (no/yes)	9358 (87.6)/1330 (12.4)
Platelet count (10^3^/μL)	154 (112–197)
Alanine aminotransferase (IU/L)	38 (24–63)
Aspartate aminotransferase (IU/L)	41 (28–62)
FIB-4 index	3.00 (1.90–5.03)
APRI	0.82 (0.47–1.58)
HCV genotype (1/2/others or multiple)^b^	7706 (72.1)/2956 (27.7)/23 (0.2)
HCV-NS5A RASs (absent/present)^c^	4118 (80.6)/989 (19.4)
Treatment regimen (DCV-ASV/LDV-SOF/OMV-PRV-Rit/EBR-GPR/DCV-ASV-BCV/SOF-RBV /OMV-PRV-Rit-RBV/GLE-PIB)	2712 (25.4)/3287 (30.8)/672 (6.3)/573 (5.4)/41 (0.4)/2415 (22.6)/117 (1.1)/871 (8.1)

Abbreviations: APRI, aspartate aminotransferase-platelet ratio index; ASV, asunaprevir; BCV, beclabuvir; DAA, direct-acting antivirals; DCV, daclatasvir; EBR, elbasvir; GLE, glecaprevir; GPR, grazoprevir; HCV, hepatitis C virus; LDV, ledipasvir; NS5A, nonstructural protein 5A; OMV, ombitasvir; PIB, pibrentasvir; PRV, paritaprevir; RAS, resistance-associated substitution; RBV, ribavirin; Rit, ritonavir; SOF, sofosbuvir.

NOTE: Values in parentheses are interquartile ranges or percentages.

^a^Cirrhosis was defined clinically by signs on imaging and endoscopic studies, including the presence of esophageal/gastric varices, collateral veins due to portal hypertension, and splenomegaly. Patients with decompensated cirrhosis were not included in this study, because the use of DAAs is not allowed in patients with decompensated cirrhosis in Japan.

^b^Including HCV genotype 3 (n = 16), genotypes 1 and 2 (n = 5), and genotypes 1 and 3 (n = 2). HCV genotype was not tested in 3 patients who received a pangenotypic glecaprevir-pibrentasvir regimen.

^c^Among 5107 patients with HCV genotype 1 infection in whom HCV-NS5A RAS status was assessed. Only amino acid residues 31 and 93 of the HCV-NS5A region were evaluated.

### Changes in Sustained Virologic Response Rates in Interferon-Free Direct-Acting Antiviral Treatment-Naive Patients and Treatment-Experienced Patients

**Figure 1.**
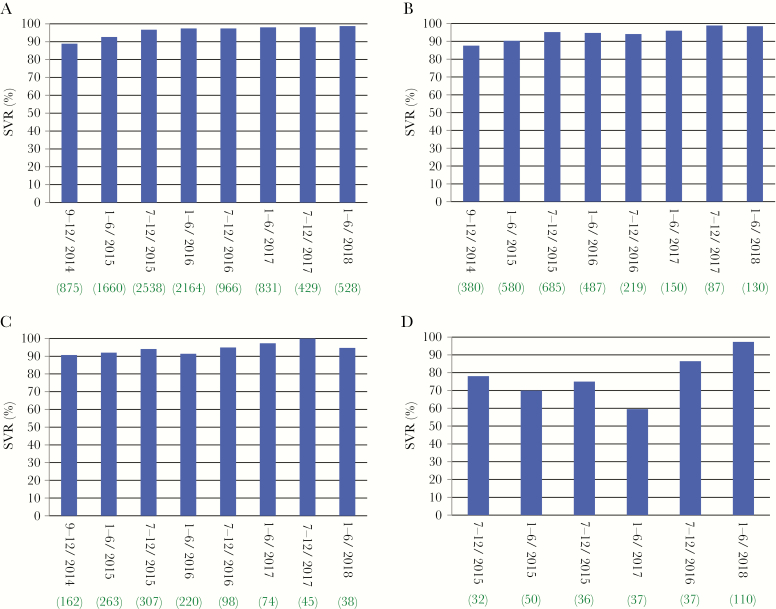
Changes in the rate of sustained virologic response (SVR) in patients over time. (A) Changes in the rate of SVR in patients with no history of interferon (IFN)-free direct-acting antiviral (DAA) therapy (n = 10 352). (B) Changes in the rate of SVR in patients with cirrhosis and no history of IFN-free DAA therapy (n = 2718). (C) Changes in the rate of SVR in patients with a history of hepatocellular carcinoma treated with curative intent and no history of IFN-free DAA therapy (n = 1207). (D) Changes in the rate of SVR in patients with a history of IFN-free DAA therapy (n = 336). Horizontal bar, the term when DAA therapy started; green number at the bottom, the number of patients in each term.

Overall, there were 9822 patients who achieved SVR (95.4%) and 471 patients who failed to achieve SVR. The SVR outcomes were not available in the remaining 395 patients due to dropout between the end of treatment and 12 weeks after treatment. There was no difference in SVR rates between participated institutions (range, 92.0%–97.4%). Figure 1A shows SVR rates by therapy start time in patients without a history of IFN-free DAA therapy. Although SVR rates were greater than 90% throughout the study period, there was an increase in SVR rates over time (*P* < .0001). This trend was maintained among patients with cirrhosis and patients with a history of HCC (both *P* < .0001) (Figure 1B and C). The SVR rates stayed above 90% after January–June 2015 and above 95% after January–June 2017, regardless of whether cirrhosis or a history of HCC was present. By contrast, SVR rates were not sufficiently high in patients with a history of IFN-free DAA therapy, ranging from 37.5% to 86.4%, except for periods when the GLE-PIB regimen was used (Figure 1D).

### Sustained Virologic Response Rates by Regimen

Table 2 shows SVR rates by regimen in patients with and without a history of IFN-free DAA therapy. In patients who have not received IFN-free DAA therapy, SVR rates were greater than 95% for all regimens except for DCV-ASV. In patients who have received IFN-free DAA therapy, only the GLE-PIB regimen had a SVR rate that was comparably high as the rate in patients who did not have a history of IFN-free DAA therapy.

**Table 2.** Sustained Virologic Response Rates by Regimen^a^

Regimen	Naive	Retreatment
Daclatasvir-asunaprevir	2441/2680 (91.1)	—
Ledipasvir-sofosbuvir	3021/3082 (98.0)	89/119 (74.8)
Ombitasvir-paritaprevir-ritonavir	638/655 (97.4)	0/2 (0)
Elbasvir-grazoprevir	519/524 (99.1)	10/16 (62.5)
Daclatasvir-asunaprevir-beclabuvir	23/23 (100)	7/18 (38.9)
Sofosbuvir-ribavirin	2259/2344 (96.4)	0/1 (0)
Ombitasvir-paritaprevir-ritonavir-ribavirin	98/102 (96.1)	9/10 (90.0)
Glecaprevir-pibrentasvir	575/581 (99.0)	133/136 (97.8)

NOTE: Percentages are given in parentheses.

^a^Daclatasvir-asunaprevir, ledipasvir-sofosbuvir, ombitasvir-paritaprevir-ritonavir, elbasvir-grazoprevir, and daclatasvir-asunaprevir-beclabuvir regimens were used for patients with hepatitis C virus (HCV) genotype 1 infection. Sofosbuvir-ribavirin and ombitasvir-paritaprevir-ritonavir-ribavirin regimens were used for patients with HCV genotype 2 infection. The glecaprevir-pibrentasvir regimen was used for patients infected with any HCV genotype.

### Factors Associated With Sustained Virologic Response

Factors associated with SVR were identified using univariate and multivariate analysis in patients infected with HCV genotypes 1 and 2. In 7706 patients with genotype 1 infection (Table 3), history of IFN-based therapy, history of IFN-free DAA therapy, presence of cirrhosis, history of HCC, DAA regimens, and presence of RASs in the HCV-NS5A region were identified as factors associated with failure to achieve SVR in the univariate analysis. Multivariate analysis showed that all of these factors were independently associated with failure to achieve SVR in patients with HCV genotype 1 infection. When focusing on 4076 patients without a history of failed IFN-free DAA therapy and no RASs, ie, 2 factors strongly associated with unfavorable treatment outcomes, univariate and multivariate analysis identified a history of IFN-based therapy, presence of cirrhosis, and DCV-ASV regimen as factors associated with failure to achieve SVR (Supplementary Table 2).

**Table 3.** Univariate and Multivariate Analysis of Baseline Factors Associated With Failure to Achieve SVR in Patients With HCV Genotype 1 Infection (N = 7706)

Factor	Category	Univariate Analysis		Multivariate Analysis	
		*P* Value	Odds Ratio (95% CI)	*P* Value	Odds Ratio (95% CI)
Age (years)		.0573		—	
Gender	Male				
	Female	.3759		—	
History of IFN-based therapy	No				
	Yes	<.0001	2.07 (1.68–2.56)	.0002	1.64 (1.27–2.13)
History of IFN-free DAA therapy	No				
	Yes	<.0001	5.40 (3.86–7.43)	<.0001	9.10 (5.26–15.76)
Cirrhosis	Absent				
	Present	<.0001	2.40 (1.95–2.97)	.0003	1.68 (1.27–2.21)
History of HCC	No				
	Yes	<.0001	1.89 (1.46–2.42)	.4775	
Regimens	GLE-PIB				
	DCV-ASV	<.0001	8.20 (3.46–26.70)	<.0001	96.10 (30.63–429.1)
	LDV-SOF	.0427	2.47 (1.03–8.13)	<.0001	12.81 (4.34–55.02)
	OMV-PRV-Rit	.0687		<.0001	36.78 (10.61–173.5)
	EBR-GPR	.3194		.0002	12.21 (3.22–59.73)
	DCV-ASV-BCV	<.0001	30.98 (9.94–117.3)	<.0001	26.30 (5.95–143.8)
HCV-NS5A-RAS^a^	Absent				
	Present	<.0001	2.94 (2.27–3.78)	<.0001	5.18 (3.72–7.19)

Abbreviations: ASV, asunaprevir; BCV, beclabuvir; CI, confidence interval; DAA, direct-acting antivirals; DCV, daclatasvir; EBR, elbasvir; GLE, glecaprevir; GPR, grazoprevir; HCC, hepatocellular carcinoma; HCV, hepatitis C virus; IFN, interferon; LDV, ledipasvir; NS5A, nonstructural protein 5A; OMV, ombitasvir; PIB, pibrentasvir; PRV, paritaprevir; RAS, resistance-associated substitution; Rit, ritonavir; SOF, sofosbuvir; SVR, sustained virologic response.

^a^Among 5107 patients with HCV genotype 1 in whom HCV-NS5A RAS was measured. Only amino acid residues 31 and 93 of the HCV-NS5A region were evaluated.

In 2956 patients with HCV genotype 2 infection (Table 4), univariate analysis identified patient age, presence of cirrhosis, a history of HCC, and DAA regimen (SOF-RBV and OMV-PRV-Rit-RBV) as factors associated with failure to achieve SVR. In multivariate analysis, all of these factors except for patient age were identified as independent factors associated with unfavorable outcome.

**Table 4.** Univariate and Multivariate Analysis of Baseline Factors Associated With Failure to Achieve SVR in Patients With HCV Genotype 2 (N = 2956)

Factor	Category	Univariate Analysis		Multivariate Analysis	
		*P* Value	Odds Ratio (95% CI)	*P* Value	Odds Ratio (95% CI)
Age (years)		.0273	1.02 (1.00–1.04)	.3735	
Gender	Male				
	Female	.9557		—	
History of IFN-based therapy	No				
	Yes	.0802		—	
History of IFN-free DAA therapy	No				
	Yes	.6667		—	
Cirrhosis	Absent				
	Present	.0005	2.24 (1.44–3.43)	.0458	1.69 (1.01–2.78)
History of HCC	No				
	Yes	.0002	3.06 (1.77–5.06)	.0093	2.29 (1.23–4.10)
Regimens	GLE-PIB				
	SOF-RBV	.0013	4.43 (1.65–18.13)	.0006	4.83 (1.79–19.81)
	OMV-PRV-Rit-RBV	.0193	5.50 (1.33–27.15)	.0085	6.96 (1.66–34.66)

Abbreviations: CI, confidence interval; DAA, direct-acting antivirals; GLE, glecaprevir; HCC, hepatocellular carcinoma; IFN, interferon; OMV, ombitasvir; PIB, pibrentasvir; PRV, paritaprevir; RBV, ribavirin; Rit, ritonavir; SOF, sofosbuvir; SVR, sustained virologic response.

### Influences of Cirrhosis or a History of Hepatocellular Carcinoma on Sustained Virologic Response in Comparison With Exact Matching

We conducted exact matching to evaluate the influence of cirrhosis on treatment outcomes. We matched 2897 patients with cirrhosis and 7791 patients without cirrhosis by age, gender, history of IFN-based therapy, history of IFN-free DAA therapy, history of HCC, HCV genotype (1 vs 2), DAA regimen, and NS5A-RAS status (wild-type, mutant, or unknown). After exact matching, each group had 1831 patients (Supplementary Figure 8). As shown in Supplementary Table 3, background characteristics were exactly matched between patients with and without cirrhosis. After matching, the SVR rate was significantly higher in patients without cirrhosis than in patients with cirrhosis (96.4% vs 93.7%, *P* = .0002).

Exact matching was also conducted to evaluate the influence of a history of HCC before IFN-free DAA therapy on the treatment outcomes. We matched 1330 patients with a history of HCC and 9358 patients with no history of HCC by age, gender, history of IFN-based therapy, history of IFN-free DAA therapy, presence of cirrhosis, HCV genotype, DAA regimen, and NS5A-RAS status. After exact matching, each group had 883 patients (Supplementary Figure 9). As shown in Supplementary Table 4, background characteristics were exactly matched between patients with and without a history of HCC. Despite a history of HCC, 34.5% of patients did not have evident cirrhosis, due to the high proportion of elderly patients with HCV infection in Japan. Hepatocellular carcinoma often develops in the absence of evident cirrhosis in this patient population [30]. After matching, there were no differences in the SVR rate between patients with no history of HCC and patients with a history of HCC (94.7% vs 93.0%, *P* = .1620).

## DISCUSSION

This study provides real-world data on IFN-free DAA therapy from a nationwide, multicenter study in Japan with more than 10 000 patients. Although the results of preapproval clinical trials were reported for each regimen before national approval and practical use in real-world settings, it is important to evaluate postapproval, real-world results for these regimens. In Japan, because all people were included in the national public insurance system and patients can undergo anti-HCV DAA therapy with low cost by a national support system for viral hepatitis treatment, the results should not be biased by socioeconomic factors. A prominent feature of patients with HCV infection in Japan is older age and high prevalence of patients with cirrhosis or those with HCC [15, 16]. Hepatitis C virus genotype is predominantly 1b, followed by 2a and 2b; other HCV genotypes and sub-genotypes are very rare in Japan [15]. The background characteristics of the study patients are reflective of characteristics of patients with HCV infection in Japan.

With the use of DAA therapy in the real-world setting, patients became younger and the prevalence of patients with cirrhosis, advanced fibrosis, or a history of HCC decreased. These trends indicate that in the first few years after IFN-free DAA therapy became available, patients who were resistant to IFN-based therapy or ineligible or intolerant of IFN-based therapy underwent IFN-free DAA therapy. By contrast, patients with newly diagnosed HCV infection predominantly underwent therapy later. Indeed, the percentage of patients with a history of IFN-based therapy decreased over time.

Although SVR rates were very high relative to rates with IFN-based therapy throughout the study period, a gradual increase in the rate of SVR was observed over time. This might be mainly due to the emergence of more effective DAA regimens, in addition to a decrease in the number of patients with factors reported to be associated with failure to achieve SVR. This trend was maintained in subgroups of patients with cirrhosis or those with a history of HCC. In addition, only DCV-ASV regimen was available for patients with HCV genotype 1 between September 2014 and September 2015 in Japan, but some patients underwent DCV-ASV regimen during this period despite the presence of NS5A-RASs, due to high risk of development of HCC or decompensation. This could contribute to the relatively lower SVR rates during this period.

Factors that are known to be strongly associated with failure to achieve SVR such as a history of failure with previous IFN-free DAA therapy and baseline HCV-NS5A-RASs were identified in univariate and multivariate analysis of virologic outcomes. In addition, cirrhosis was identified as a factor associated with failure to achieve SVR. By contrast, a history of HCC was not a factor associated with failure to achieve SVR overall, although it was identified as a factor associated with failure to achieve SVR in patients with HCV genotype 2 infection.

We conducted exact matching of patients and compared SVR rates between patients with and without cirrhosis and between patients with and without a history of HCC to accurately evaluate the effects of these factors. The large number of study patients enabled exact matching. Unlike propensity score matching, the factors used for matching were exactly the same between the 2 groups. Although there are several controversial reports on the effect of a history of HCC [31, 32], the comparison between patients with no history of HCC and those with a history of HCC in this study did not show a difference in SVR rates. By contrast, there was a significant difference in SVR rates based on the presence or absence of cirrhosis. Exact matching and multivariate analysis showed that cirrhosis is a host factor significantly associated with failure to achieve SVR, although the difference was small. Most preapproval clinical trials reported that patients with and without cirrhosis had comparable rates of SVR in Japan and around the world [33]. By contrast, several studies based on real-world data on specific DAA regimens reported lower SVR rates in patients with cirrhosis compared with that in patients without cirrhosis [34–37]. The result of this study strongly supports that the presence of cirrhosis unfavorably influences the achievement of SVR across regimens, even when cirrhosis is compensated.

There are several limitations to this study. This is a descriptive study on real-world use of DAA regimens, and selection of regimens was not perfectly based on Japanese guidelines of DAA therapy for HCV that frequently updated during the study period. However, the results reflected real-world treatment situation during the period. The study patients did not include patients with decompensated cirrhosis or active HCC, because IFN-free DAA therapy is not approved by the national insurance system in Japan for patients with these conditions during the study period. Therefore, we only evaluated the effects of compensated cirrhosis and HCC treated with curative intent. Further studies will be necessary to understand the effects of decompensated cirrhosis or active HCC on SVR rates. The analysis of RASs was focused on only amino acid residues 31 and 93 of the HCV-NS5A regions in patients with HCV genotype 1 infection. There are reportedly several other RASs not only in the NS5A region but also in the NS3 or NS5B regions, although these are very rare at baseline in patients with no history of IFN-free DAA therapy. In this large multicenter study, it was difficult to obtain detailed information on RASs for all patients. No data on RASs in patients with HCV genotype 2 infection and a considerable proportion of patients with HCV genotype 1 infection were available. Regarding host factors, no data on baseline renal function or hemoglobin levels were available. In addition, this study focused on the efficacy of the therapy, and the data on adherence or tolerability were not documented. Finally, the number of patients who received each regimen varied. However, despite of these limitations, this study had a large number of patients and provided solid results on factors associated with SVR.

## CONCLUSIONS

In conclusion, this nationwide, multicenter study from Japan with more than 10 000 patients showed changes in background characteristics of patients with HCV infection undergoing IFN-free DAA therapy. Screening for identifying patients with HCV infection for anti-HCV therapy should be enhanced in the future, given the high virologic efficacy of the current DAA regimen. In addition to baseline RASs and a history of previous DAA therapy failure, cirrhosis is a factor associated with IFN-free DAA therapy failure, whereas a history of HCC is not associated with treatment outcome.

## Supplementary Data

Supplementary materials are available at *Open Forum Infectious Diseases* online. Consisting of data provided by the authors to benefit the reader, the posted materials are not copyedited and are the sole responsibility of the authors, so questions or comments should be addressed to the corresponding author.

ofz185_suppl_supplementary_material

ofz185_suppl_supplementary_tables_figures
