# Supplementary material for: Trends and Efficacy of Interferon-Free Anti–hepatitis C Virus Therapy in the Region of High Prevalence of Elderly Patients, Cirrhosis, and Hepatocellular Carcinoma: A Real-World, Nationwide, Multicenter Study of 10 688 Patients in Japan
Source: Open Forum Infect Dis. 2019 Apr 15;6(5):ofz185. doi: 10.1093/ofid/ofz185 (PMC6524830; doi:10.1093/ofid/ofz185)
Supplement: ofz185_suppl_supplementary_material [file ofz185_suppl_supplementary_material.docx]

**Supplementary Text**

**Results**

*Changes in background characteristics of patients who underwent IFN-free DAA therapy*

Supplementary Fig. 2 shows the change in the number of patients by HCV genotype based on when the treatment started by 3-month interval starting with September-December 2014. Arrows indicate the time points when regimens were approved. The number of patients increased after the approval of the first DAA regimen (DCV-ASV), peaked during October–December 2015, and decreased afterwards. Supplementary Figs. 3 and 4 shows changes in baseline age and laboratory liver fibrosis indices (FIB-4 index and APRI) of patients by when therapy started by 6-month interval. Patient age, FIB-4 index, and APRI values decreased, suggesting that the percentage of patients with milder liver fibrosis increased over time (all *p*<0.0001). Accordingly, the percentage of patients with cirrhosis decreased (Supplementary Fig. 5, *p*<0.0001), except for a small increase after the approval of the GLE-PIB regimen. In addition, the percentage of patients with a history of HCC decreased (Supplementary Fig. 6, *p*<0.0001). The percentage of patients with a history of IFN-based therapy also decreased, from more than 50% during September–December 2014 to around 10% during the first half of 2018 (Supplementary Fig. 7, *p*<0.0001).
