# Supplementary material for: Trends and Efficacy of Interferon-Free Anti–hepatitis C Virus Therapy in the Region of High Prevalence of Elderly Patients, Cirrhosis, and Hepatocellular Carcinoma: A Real-World, Nationwide, Multicenter Study of 10 688 Patients in Japan
Source: Open Forum Infect Dis. 2019 Apr 15;6(5):ofz185. doi: 10.1093/ofid/ofz185 (PMC6524830; doi:10.1093/ofid/ofz185)
Supplement: ofz185_suppl_supplementary_tables_figures [file ofz185_suppl_supplementary_tables_figures.doc]

Supplementary Table 1. Number of patients with prior DAA therapy that failed to achieve SVR (N = 336)

| Regimen | Number of patients (%) |
| --- | --- |
| HCV genotype 1 |  |
| Daclatasvir–asunaprevir | 198 (58.9) |
| Ledipasvir–sofosbuvir | 41 (12.2) |
| Ombitasvir–paritaprevir–ritonavir | 12 (3.6) |
| Elbasvir–grazoprevir | 3 (0.9) |
| Daclatasvir–asunaprevir and ledipasvir–sofosbuvir | 15 (4.5) |
| Daclatasvir–asunaprevir and elbasvir–grazoprevir | 6 (1.8) |
| Daclatasvir–asunaprevir and daclatasvir–asunaprevir–beclabuvir | 2 (0.6) |
| Ombitasvir–paritaprevir–ritonavir and elbasvir–grazoprevir | 1 (0.3) |
| Daclatasvir–asunaprevir, ledipasvir–sofosbuvir, and daclatasvir–asunaprevir–beclabuvir | 1 (0.3) |
| Daclatasvir–asunaprevir, ledipasvir–sofosbuvir, and elbasvir–grazoprevir | 1 (0.3) |
| HCV genotype 2 |  |
| Sofosbuvir–ribavirin | 49 (14.6) |
| Ombitasvir–paritaprevir–ritonavir–ribavirin | 7 (2.1) |

Supplementary Table 2. Univariate and multivariate analysis of baseline factors associated with failure to achieve SVR in patients with HCV genotype 1 infection and no history of previous IFN-free DAA therapy and no baseline RASs in the HCV-NS5A region (N = 4,076)

| Factor |  | Univariate analysis | | Multivariate analysis | | |
| --- | --- | --- | --- | --- | --- | --- |
|  |  | *p* value | Odds ratio  (95% CI) | *p* value | | Odds ratio  (95% CI) |
| Age (years) |  | 0.5944 |  | | ----- |  |
| Gender | Male |  |  | |  |  |
|  | Female | 0.1338 |  | | ----- |  |
| History of IFN-based therapy | No |  |  | |  |  |
|  | Yes | <0.0001 | 1.98 (1.44–2.72) | | 0.0147 | 1.50 (1.08–2.08) |
| Cirrhosis* | Absent |  |  | |  |  |
|  | Present | <0.0001 | 2.02 (1.46–2.77) | | 0.0052 | 1.60 (1.15–2.22) |
| History of HCC | No |  |  | |  |  |
|  | Yes | 0.6482 |  | | ----- |  |
| Regimens | GLE-PIB |  |  | |  |  |
|  | DCV-ASV | 0.0035 | 4.93 (1.55–30.03) | | 0.0129 | 4.13 (1.29–25.24) |
|  | LDV-SOF | 0.1964 |  | | 0.1759 |  |
|  | OMV-PRV-Rit | 0.3818 |  | | 0.3891 |  |
|  | EBR-GPR | 0.2187 |  | | 0.2258 |  |
|  | DCV-ASV-BCV | 0.5622 |  | | 0.5831 |  |

SVR, sustained virologic response; CI, confidence interval; IFN, interferon; HCC, hepatocellular carcinoma; GLE, glecaprevir; PIB, pibrentasvir; DCV, daclatasvir; ASV, asunaprevir; LDV, ledipasvir; SOF, sofosbuvir; OMV; ombitasvir; PRV, paritaprevir; Rit, ritonavir; EBR, elbasvir; GPR, grazoprevir; BCV, beclabuvir.

Supplementary Table 3. Background characteristics of patients with and without cirrhosis selected by exact matching (N = 3,662)

| Factors | Patients without cirrhosis  (n = 1,831) | Patients with cirrhosis  (n = 1,831) | *P* value |
| --- | --- | --- | --- |
| Age (years) | 71 (26–88) | 71 (26–88) | 1.000 |
| Gender (male / female) | 845 (46.1) / 986 (53.9) | 845 (46.1) / 986 (53.9) | 1.000 |
| History of interferon-based therapy (no / yes) | 1307 (71.4) / 524 (28.6) | 1307 (71.4) / 524 (28.6) | 1.000 |
| History of interferon-free DAA therapy (no / yes) | 1811 (98.9) / 20 (1.1) | 1811 (98.9) / 20 (1.1) | 1.000 |
| History of HCC (no / yes) | 1658 (90.6) / 173 (9.4) | 1658 (90.6) / 173 (9.4) | 1.000 |
| Regimen |  |  | 1.000 |
| Daclatasvir–asunaprevir | 644 (35.2) | 644 (35.2) |  |
| Ledipasvir–sofosbuvir | 589 (32.2) | 589 (32.2) |  |
| Ombitasvir–paritaprevir–ritonavir | 72 (3.9) | 72 (3.9) |  |
| Elbasvir–grazoprevir | 66 (3.6) | 66 (3.6) |  |
| Daclatasvir–asunaprevir–beclabuvir | 0 | 0 |  |
| Sofosbuvir–ribavirin | 361 (19.7) | 361 (19.7) |  |
| Ombitasvir–paritaprevir–ritonavir–ribavirin | 3 (0.2) | 3 (0.2) |  |
| Glecaprevir–pibrentasvir | 96 (5.2) | 96 (5.2) |  |
| HCV genotype (1/ 2) | 1411 (77.1) / 420 (22.9) | 1411 (77.1) / 420 (22.9) | 1.000 |
| HCV-NS5A-RAS* |  |  | 1.000 |
| No (wild type) | 784 (55.6) | 784 (55.6) |  |
| Yes (mutant type) | 122 (8.6) | 122 (8.6) |  |
| Unknown | 505 (35.8) | 505 (35.8) |  |

*Only in patients with HCV genotype 1 infection.

DAA, direct-acting antivirals; HCC, hepatocellular carcinoma; HCV, hepatitis C virus; NS5A, non-structural protein 5A; RAS, resistance-associated substitution.

Supplementary Table 4. Background characteristics of patients with and without a history of HCC selected by exact matching (N = 1,766)

| Factors | Patients with no history of HCC (n = 883) | Patients with a history of  HCC (n = 883) | *P* value |
| --- | --- | --- | --- |
| Age (years) | 73 (40–88) | 73 (40–88) | 1.000 |
| Gender (male / female) | 491 (55.6) / 392 (44.4) | 491 (55.6) / 392 (44.4) | 1.000 |
| History of interferon-based therapy (no / yes) | 612 (69.3) / 271 (30.7) | 612 (69.3) / 271 (30.7) | 1.000 |
| History of interferon-free DAA therapy (no / yes) | 876 (99.2) / 7 (0.8) | 876 (99.2) / 7 (0.8) | 1.000 |
| Cirrhosis (no / yes) | 305 (34.5) / 578 (65.5) | 305 (34.5) / 578 (65.5) | 1.000 |
| Regimen |  |  | 1.000 |
| Daclatasvir–asunaprevir | 332 (37.6) | 332 (37.6) |  |
| Ledipasvir–sofosbuvir | 288 (32.6) | 288 (32.6) |  |
| Ombitasvir–paritaprevir–ritonavir | 33 (3.7) | 33 (3.7) |  |
| Elbasvir–grazoprevir | 26 (2.9) | 26 (2.9) |  |
| Daclatasvir–asunaprevir–beclabuvir | 0 | 0 |  |
| Sofosbuvir–ribavirin | 175 (19.8) | 175 (19.8) |  |
| Ombitasvir–paritaprevir–ritonavir–ribavirin | 1 (0.1) | 1 (0.1) |  |
| Glecaprevir–pibrentasvir | 28 (3.2) | 28 (3.2) |  |
| HCV genotype (1/ 2) | 690 (78.1) / 193 (21.9) | 690 (78.1) / 193 (21.9) | 1.000 |
| HCV-NS5A-RAS* |  |  | 1.000 |
| No (wild type) | 389 (56.4) | 389 (56.4) |  |
| Yes (mutant type) | 57 (8.3) | 57 (8.3) |  |
| Unknown | 244 (35.3) | 244 (35.3) |  |

*Only in patients with HCV genotype 1 infection.

HCC, hepatocellular carcinoma; DAA, direct-acting antivirals; HCV, hepatitis C virus; NS5A, non-structural protein 5A; RAS, resistance-associated substitution.

Supplementary figure 1. Location of participating institutions

Supplementary figure 2. Changes in the number of patients who started interferon-free DAA therapy by genotype and regimen approval date (green arrows). A, daclatasvir–asunaprevir (for genotype 1); B, sofosbuvir–ribavirin (for genotype 2); C, ledipasvir–sofosbuvir (for genotype 1); D, ombitasvir–paritaprevir–ritonavir (for genotype 1); E, ombitasvir–paritaprevir–ritonavir–ribavirin (for genotype 2); F, elbasvir–grazoprevir (for genotype 1); G, daclatasvir–asunaprevir–beclabuvir (for genotype 1); H, glecaprevir–pibrentasvir (for all genotypes).

Supplementary figure 3. Changes in age of patients who started interferon-free DAA therapy.

Blue line, median age; Red line, mean age.

Supplementary figure 4. Changes in baseline laboratory liver fibrosis indices.

Blue line, median FIB-4 index; red line, median aspartate aminotransferase–platelet ratio index.

Supplementary figure 5. Changes in the percentage of patients with cirrhosis

Supplementary figure 6. Changes in the percentage of patients with a history of hepatocellular carcinoma

Supplementary figure 7. Changes in the percentage of patients with a history of interferon-based therapy

Supplementary figure 8. Schematic flowchart of the exact matching for the presence of cirrhosis

Supplementary figure 9. Schematic flowchart of the exact matching for the history of HCC
